# Altered Inhibitory Mechanisms in Parkinson’s Disease: Evidence From Lexical Decision and Simple Reaction Time Tasks

**DOI:** 10.3389/fnhum.2021.624026

**Published:** 2021-04-26

**Authors:** Alban Letanneux, Jean-Luc Velay, François Viallet, Serge Pinto

**Affiliations:** ^1^University Paris Est Creteil, CHArt, Bonneuil, France; ^2^UPL, University Paris 8, CHArt, Saint-Denis, France; ^3^EPHE, PSL University, CHArt, Aubervilliers, France; ^4^Aix-Marseille Univ, CNRS, LNC, Marseille, France; ^5^Aix-Marseille Univ, CNRS, LPL, Aix-en-Provence, France; ^6^Neurology Department, Centre Hospitalier du Pays d’Aix, Aix-en-Provence, France

**Keywords:** Parkinson’s disease, lexical access, inhibition capability, language, cognitive impairment

## Abstract

**Introduction:**

Although the motor signs of Parkinson’s disease (PD) are well defined, nonmotor symptoms, including higher-level language deficits, have also been shown to be frequent in patients with PD. In the present study, we used a lexical decision task (LDT) to find out whether access to the mental lexicon is impaired in patients with PD, and whether task performance is affected by bradykinesia.

**Materials and Methods:**

Participants were 34 nondemented patients with PD, either without (*off*) medication (*n* = 16) or under optimum (*on*) medication (*n* = 18). A total of 19 age-matched control volunteers were also recruited. We recorded reaction times (RTs) to the LDT and a simple RT (control) task. In each task, stimuli were either visual or auditory. Statistical analyses consisted of repeated-measures analyses of variance and Tukey’s HSD *post hoc* tests.

**Results:**

In the LDT, participants with PD both *off* and *on* medication exhibited intact access to the mental lexicon in both modalities. In the visual modality, patients *off* medication were just as fast as controls when identifying real words, but slower when identifying pseudowords. In the visual modality of the control task, RTs for pseudowords were significantly longer for PD patients *off* medication than for controls, revealing an unexpected but significant lexicality effect in patients that was not observed in the auditory modality. Performances of patients *on* medication did not differ from those of age-matched controls.

**Discussion:**

Motor execution was not slowed in patients with PD either *off* or *on* medication, in comparison with controls. Regarding lexical access, patients *off* medication seemed to (1) have difficulty inhibiting a cognitive-linguistic process (i.e., reading) when it was not required (simple reaction time task), and (2) exhibit a specific pseudoword processing deficit in the LDT, which may have been related to impaired lateral word inhibition within the mental lexicon. These deficits seemed to be compensated by medication.

## Introduction

Although the motor symptoms of Parkinson’s disease (PD) are well defined and described, nonmotor features have been increasingly recognized in recent years as being inherent to the disease (Chaudhuri and Schapira, 2009; Zis et al., 2015). Cognitive deterioration is a common, progressive and disabling feature of PD, arising from neuropsychological, neurochemical, structural, and pathophysiological changes (Pagonabarraga and Kulisevsky, 2012). However, important questions about cognitive disorders in patients without dementia have yet to be addressed (Barone et al., 2011). Research over the past two decades on the various processes specific to language impairment in PD (for reviews, see Murray, 2008; Altmann and Troche, 2011; Colman and Bastiaanse, 2011; Auclair-Ouellet et al., 2017) indicates that language disorders should be viewed as part of the spectrum of cognitive deficits in patients with PD without dementia, as also recommended by the Movement Disorder Society task force on cognitive impairment (Litvan et al., 2011). For example, higher-level language processes have been shown to be impaired in patients with PD, affecting various aspects of language comprehension such as complex sentence structure understanding (Lieberman et al., 1992; Lee et al., 2003; Hochstadt et al., 2006; Angwin et al., 2006a), metaphor and ambiguous sentence comprehension (Berg et al., 2003; Monetta and Pell, 2007), inference generation (Monetta et al., 2008), and irony comprehension (Monetta et al., 2009). In terms of language production, individuals with PD appear to produce mainly simple sentences (Illes et al., 1988; Murray, 2000; Murray and Lenz, 2001), punctuated by numerous pauses and presenting many acoustic variations associated with motor speech difficulties (Illes et al., 1988; Illes, 1989). Whether these deficits are caused by a language-specific impairment or more general deficits in other cognitive functions, such as executive functioning or working memory, is an ever present issue for researchers (Lee et al., 2003; Longworth et al., 2005; Terzi et al., 2005; Angwin et al., 2006b; Hochstadt et al., 2006).

One of these higher-level language processes is word recognition, which is commonly tested. Word recognition can be estimated by measuring access to the mental lexicon^1^, classically by using a lexical decision task (LDT; Moret-Tatay and Perea, 2011). Participants have to decide as quickly as possible whether a word (auditory or visual presentation) is a real word or not (i.e., a pseudoword). The response (i.e., manual button press) is faster for a word than for a pseudoword, and the time difference can be interpreted as the result of a *lexicality effect*, also called *word superiority effect* (e.g., Cattell, 1886; Henderson, 1982). According to psycholinguistic models of lexical access (e.g., Coltheart et al., 2001; Hauk et al., 2006), word recognition can be viewed as a series of processes occurring in cascade, where lexical access precedes meaning processing. In LDTs, with visual stimuli, it is commonly assumed that word/pseudoword reading involves two parallel and complementary routes: a direct, lexical (lexicosemantic) route, and an indirect, sublexical (phonological) one (Coltheart, 1978). Roughly speaking, the lexical pathway affords direct access to orthographic information about the words, and thence to the semantic network. This route makes it possible to recognize visually familiar words, but it is less helpful for visually deciphering unfamiliar words, including pseudowords. It is therefore the indirect sublexical pathway that underlies the process of connecting the orthographic and phonological features of unfamiliar words or pseudowords, allowing them to be read. The use of this circuit relies on the segmentation of words into graphemes, and then the matching of these graphemes with their related phonemes. As the name of this route implies, words are identified according to their phonological coding. This dual-route model of visual word recognition^2^ was inspired by the interactive activation (IA) model (McClelland and Rumelhart, 1981), which itself was based on a concept put forward by Morton (1979). According to the IA model, written word recognition involves three levels of parallel processing: (1) visual features (e.g., horizontal, vertical, and diagonal lines), (2) letters, and (3) words. Letters are coded according to their position within the word and processed simultaneously. The different units are interconnected within and between levels. The connections are excitatory between two compatible units, and inhibitory between two incompatible units. At the word level, there is a mechanism of mutual lexical inhibition of all active lexical candidates, to allow for recognition of the target word. This is commonly referred to as *lateral inhibition*. In the same vein, based on the concept of spreading activation (McClelland and Rumelhart, 1981), auditory word recognition can be interpreted according to the TRACE model (McClelland and Elman, 1986). It again involves subsystems processed in parallel, with three distinct levels: (1) acoustic features (e.g., intensity, timbre, duration, and pitch), (2) phonemes, and (3) words. Acoustic information activates phonemic representations containing the acoustic characteristics, which in turn activate words that contain them (lexical entries) in the right order. This takes place automatically, while the acoustic information is being processed. Each processing level is linked by excitatory connections to other levels, and the selection of the word to be recognized is made possible by inhibitory links between competing units, where the most active unit (i.e., the one most compatible with the perceived acoustic features) inhibits the less active ones. Together, the IA (McClelland and Rumelhart, 1981) and TRACE (McClelland and Elman, 1986) models predict direct access to the mental lexicon during the visual or auditory presentation of words. This explains the faster recognition of words compared with pseudowords, subtended by the pairing between the signal and the information contained in the mental lexicon. Here again, this process is strongly modulated by activation and inhibition mechanisms within the processing levels, in the form of lexical competition / lateral inhibition between words. Thus, after the visual or auditory presentation of a word, different competitors sharing traits with the target word are automatically activated. Lateral inhibition between these competitors allows those with the highest activation levels, including the target word, to predominate and eliminate those with a lower activation level. Accordingly, not only does the target word try to inhibit its competitors, but the latter also send inhibitory information to the target word (Dufour and Frauenfelder, 2007).

Some versions of the LDT feature semantic priming. This involves enhancing lexical access by presenting a semantically related word prime beforehand, in order to test structural/functional connections between words in the mental lexicon. The result is faster recognition when the word is preceded by a semantically related prime word (e.g., apple-fruit) rather than by an unrelated one (e.g., paper-fruit). Phonological (auditory presentation of stimuli) and orthographic (visual presentation of stimuli) priming can be used to test other levels of connections between words. Lexical access has seldom been investigated in patients with PD, and most studies have focused on the processes underlying access to semantic representations in these patients (Hines and Volpe, 1985; Spicer et al., 1994; McDonald et al., 1996; Copland, 2003; Filoteo et al., 2003; Angwin et al., 2005; Marí-Beffa et al., 2005; Ito and Kitagawa, 2006; Pederzolli et al., 2008; Ehlen et al., 2013). LDT with semantic priming has often been performed for this purpose. The very first study among patients with PD reported that the amplitude of the semantic priming effect was comparable to that achieved by healthy individuals (Hines and Volpe, 1985). Patients had longer reaction times (RTs) than controls when primes were unrelated, but not when they were semantically related (Spicer et al., 1994; McDonald et al., 1996), leading to the so-called *hyperpriming concept*, which has sometimes come in for criticism for methodological reasons (Arnott and Chenery, 1999, 2001). This hyperpriming could be regarded as part of the semantic processing deficits in PD (Copland, 2003), possibly caused by the abnormal persistence of lexical activation of primes in memory (Filoteo et al., 2003), or poor inhibition of irrelevant information from distractors (Angwin et al., 2005; Marí-Beffa et al., 2005). These findings point to the involvement of the basal ganglia in the facilitation and inhibition processes. The semantic priming effects observed in patients with or without dopaminergic treatment suggest that dopamine depletion leads to both a lower level of activation during automatic semantic processing, and a decrease in the intensity of this activation in the semantic network, restored by the medication (Copland et al., 2000; Arnott et al., 2001, 2011; Angwin et al., 2006b, 2009; Castner et al., 2007). Therefore, altered semantic activation in patients with PD seems to stem from dopamine loss. Furthermore, it should be noted that most studies so far have featured visual stimuli, with only a few investigations considering the auditory presentation modality (e.g., Copland, 2003; Ehlen et al., 2013). There have been even fewer studies using phonological priming (e.g., Elorriaga-Santiago et al., 2012).

In the present study, we explored lexical access by investigating the lexicality effect with an LDT task without any priming (i.e., semantic, phonological, or orthographic) in participants with PD and age-matched controls. This version of the LDT appeared to be the most appropriate one, as we wanted to study lexical access *per se*, rather than enhance it. Our first objective was to determine whether the motor execution of the task used to measure lexical access was affected by bradykinesia. Our second objective was to look for differences and similarities in responses to the visual vs. auditory stimuli in the LDT, in order to identify the mechanisms of lexical access in patients with PD and in these two modalities.

## Materials and Methods

### Participants

Participants were 34 nondemented patients with PD, either without (*off*; *n* = 16; mean age = 66.6 years, and *SD* = 7.4), or under optimum (*on*; *n* = 18; mean age = 65.1 years, and *SD* = 7.1) medication. The patients attended the Neurology Department of Aix-en-Provence Hospital (France). They met all the Parkinson’s UK Brain Bank criteria (Hughes et al., 1992) for the diagnosis of idiopathic PD. Dopaminergic denervation was objectified by striatal dopamine transporter visualization using single-photon emission computed tomography (ioflupane iodine-123 injection, DaTscan^TM^). Patients were not recruited if they had any history of stroke, depression, impulse control disorder, drug or alcohol abuse, as this might have interfered with their ability to perform the tasks. Patients with deep brain stimulation were also excluded. Levodopa equivalence daily dose (LEDD) was calculated according to standard formulae (Tomlinson et al., 2010; Schade et al., 2020). Motor disability was assessed with Part III (Items 18–31) of the Unified Parkinson’s Disease Rating Scale (UPDRS; Fahn et al., 1987), and cognitive impairment with the Mattis Dementia Rating Scale (MDRS; Mattis, 1988). As recommended by Llebaria et al. (2008), the MDRS cut-off score was set at 123/144 for the screening of dementia in patients.

A third group of participants consisted of 19 age-matched controls (mean age = 65.2 years, *SD* = 5.6 years), recruited via a call for participation and with the same exclusion criteria as for patients. Table 1 summarizes participants’ characteristics.

**TABLE 1:** Participants’ demographic and clinical data.

	Patients *off*	Patients *on*	Controls
	medication	medication	
Sex, M/F	6/10	10/8	9/10
Mean age in years ± *SD*	66.6 ± 7.4	65.1 ± 7.1	65.2 ± 5.6
MDRS mean score ± *SD* (/144)	136.5 ± 3.7	137.2 ± 4.9	141.5 ± 2.4
Mean disease duration in years ± *SD*	6.81 ± 4.4	5.4 ± 4.2	n/a
UPDRS III mean score ± *SD* (/108)	21.8 ± 8.6	11.7 ± 6.1	n/a
Mean LEDD (mg/day) ± *SD*	811.4 ± 385.9	805.0 ± 431	n/a

*LEDD, levodopa equivalent daily dose; MDRS, Mattis Dementia Rating Scale; M/F, Male/Female; UPDRS III, Motor Part (Items 18–31) of the Unified Parkinson’s Disease Rating Scale; and *SD*, Standard-deviation.*

All participants were right-handed (Edinburgh Handedness Inventory >75%; Oldfield, 1971) and native French speakers. They had normal or corrected-to-normal vision and self-reported normal-for-age hearing. The study was approved by the local institutional review board (Ethical Research Committee Sud Méditerranée 1, protocol no. 12 42). In accordance with the Declaration of Helsinki (World Medical Association, 2001), all participants provided their written informed consent.

Analyses of variance (ANOVAs) for between-group comparisons with Tukey’s HSD *post hoc* test and Bonferroni correction revealed (1) similar mean ages for participants in all three groups, *F*(2, 32) = 0.36, *p* = 0.70), (2) significantly lower MDRS scores for patients both *off* (*p*_Bonferroni_ < 0.001, Cohen’s *d* = 1.313) and *on* (*p*_Bonferroni_ < 0.001, Cohen’s *d* = 1.167) medication than controls, *F*(2, 32) = 13.4, *p* < 0.001. Welch’s *t* tests showed that neither disease duration (*t* = 0.93, *p* = 0.36, and Cohen’s *d* = 0.32) nor LEDD (*t* = 0.04, *p* = 0.96, and Cohen’s *d* = 0.014) differed between patients *off* and *on* medication. Additionally, patients *off* medication had higher UPDRS III scores (*t* = 3.70, *p* < 0.001, and Cohen’s *d* = 1.30) than patients *on* medication.

### Protocol and Stimulus Validation

Prior to running the experiment with the patients and age-matched controls, we tested and validated the stimuli we had selected (words) or created (pseudowords) with a group of 40 young adults (men/women = 20/20; mean age = 20.6 years, and *SD* = 2.3). The objective of this validation experiment was to confirm that (1) RTs for words vs. pseudowords in the simple reaction time task (SRTT) did not differ, and (2) the stimuli we used in the LDT elicited a lexicality effect. Exclusion criteria were the same as those for the patients with PD and age-matched controls.

### Experimental Design

The *off* medication group was assessed after an overnight medication fast (i.e., after 12 h without any treatment), in order to be as close as possible to the Parkinsonian state. The *on* medication group was also assessed in the morning, after the usual morning dose treatment (i.e., after 60-90 min). Participants were seated at a comfortable viewing distance from a computer screen in a quiet room at the hospital. To maximize the lexicality effect and avoid any familiarity with the items, they started the experiment with the LDT in the two modalities. The order of presentation (visual vs. auditory stimuli) was counterbalanced across participants. Participants then performed a SRTT in the two modalities, to estimate their distal motor state, as proposed by the Movement Disorder Society task force on cognitive impairment (Litvan et al., 2011). This enabled us to pinpoint the impact of motor execution on LDT performance.

For both tasks in the visual modality, the sequence of experimental trials was as follows: (a) a fixation cross (+) was displayed for 720 ms; (b) this was followed by a white screen with a random duration of 500-1,000 ms (this interstimulus interval served to maintain the participant’s attention); (c) a stimulus was displayed in the centre of the screen until the participant responded; and (d) the following trial then began automatically after 500 ms. All items were randomly presented, in black capital letters (12-point Arial font) against a white background, on a 20” CRT monitor (60 Hz).

For both tasks in the auditory modality, there was a similar sequence of trials, except that the fixation cross was replaced with a 100-ms auditory signal (beep). Words and pseudowords were played via a headset (Sennheiser PC 151; volume adjusted to each participant prior to the experiment).

For both the visual and auditory versions of the LDT, participants indicated whether the stimulus was a real word or not as quickly as possible, but without compromising accuracy, by pressing the *word* or *pseudoword* buttons of a serial response box (model 200A, Psychology Software Tools) with the index or middle finger of their right hand. To avoid any possible difference in movement initiation latency between the two fingers (Wilimzig et al., 2012), the associations between response buttons and fingers were counterbalanced, as is commonly done (Fernandino et al., 2013).

For the SRTT, participants had to press a button of the response pad as quickly as possible whenever a visual or auditory stimulus was presented (i.e., immediately after stimulus onset), with the index (for half the trials, *n* = 10), or middle finger (for the remaining trials, *n* = 10). Three lists of 20 stimuli randomly extracted from the original set were used in this task, counterbalanced across participants.

In both the visual and auditory modalities, the tasks were preceded by four practice trials. RTs were digitally recorded by dedicated software (E-Prime^®^, Psychology Software Tools), starting from the onset of the stimulus.

### Stimuli

We selected 30 five-letter, bisyllabic words with the same CVCVC (C: consonant; V: vowel) phonological and orthographic pattern from a French database (Lexique, v3.71; New et al., 2001). No other characteristics (e.g., frequency, lexical neighborhood) of these items could be controlled (see Supplementary Material). We also constructed 30 orthographically legal and pronounceable pseudowords. In order to match these pseudowords with the real words as closely as possible, in terms of number of letters and bigram frequency, we generated them using syllabic segmentation: the second syllable of one selected word was randomly associated with the first syllable of another selected word, taking care to avoid contructing a real French word (e.g., the real words *lapin* [rabbit] and *melon* [melon] could be used to create the pseudowords *lalon* and *mepin*).

Auditory stimuli were recorded in a soundproof room by a trained native French speaker, and were then segmented and preprocessed (Praat software, version 3.5.05; Boersma and Weenink, 2009). The words (498 ± 52 ms) and pseudowords (510 ± 64.3 ms) did not differ significantly on duration (Welch’s *t* test, *t* = -0.77, *ns*).

These stimuli were used in both experimental tasks (LDT and SRTT) in both sensory modalities (visual and auditory).

### Statistical Analyses

We only analyzed RTs for correct trials. All temporal errors were removed from analyses (i.e., RTs below 200 ms or above 3,000 ms for the two LDTs, and RTs below 100 ms or above 1,500 ms for the two SRTTs). Following this preprocessing, individual and group outliers (defined as any RT more than two *SD*s above or below the mean) were also excluded from the analyses. This procedure ensured that the results were not driven by a small number of atypical data points (Ratcliff, 1993). In the visual modality, errors (control group = 3.07%; PD group = 4.06%), and outlier RTs resulted in the removal of a total of 9.05% of the dataset for the LDT, and 9.03% for the SRTT. In the auditory modality, errors (controls = 5.09%, PD group = 8.96%) and outlier RTs resulted in the removal of 11.57% of the dataset for the LDT, and 6.60% for the SRTT.

Two separate repeated-measures ANOVAs with group (control vs. PD *off* and control vs. PD *on*) as a between-groups factor and lexicality (words, pseudowords) as a within-participants factor were performed on RTs. They were conducted with participants (*F*_1_) and items (*F*_2_) as random variables. Another ANOVA with group (control vs. PD *off* vs. PD *on*) as between-group factor and lexicality (words, pseudowords) as within-subject factor has been performed on number of errors for both LDTs. Estimated effect sizes are reported as partial eta squared (η^2^_p_; Lakens, 2013; Wasserstein and Lazar, 2016). Tukey HSD *post hoc* comparisons were also performed when appropriate, with Bonferroni correction (Zar, 1984) for multiple comparisons. The statistical significance level was set at *p* ≤ 0.05. The data were preprocessed in the RStudio environment (v. 0.99.484), implementing R software (v.3.2.2; R Development Core Team, 2014), and analyses were performed using Jamovi (version 1.2.27; The Jamovi Project, 2020).

## Results

### Stimulus Validation

For the LDT in the visual modality, RTs were significantly shorter for words than for pseudowords (572 ± 73 ms vs. 646 ± 86 ms), *F*_1_(1, 39) = 53.5, *p* < 0.001, and η^2^*_p_* = 0.578 and *F*_2_(1, 58) = 53.4, *p* < 0.001, and η^2^*_p_* = 0.479. In the auditory modality, RTs were also shorter for words (mean = 801 ± 73 ms) than for pseudowords (mean = 871 ± 113 ms), *F*_1_(1, 39) = 71.9, *p* < 0.001, and η^2^*_p_* = 0.648 and *F*_2_(1, 58) = 46.8, *p* < 0.001, and η^2^*_p_* = 0.446.

For the SRTT, mean RT was 220 ms (±26) for both words and pseudowords in the visual modality, and 267 ms (±68) for both words and pseudowords in the auditory modality. No lexicality effect was observed in either the visual, *F*_1_ (1, 39) = 0.1, *p* = 0.78, and η^2^*_p_* = 0.002 and *F*_2_(1, 58) = 0.15, *p* = 0.90, and η^2^*_p_* = 0.000, or auditory modality, *F*_1_(1, 39) = 0.2, *p* = 0.87, and η^2^*_p_* = 0.001 and *F*_2_(1, 58) = 0.0, *p* = 0.98, and η^2^*_p_* = 0.000.

### Lexical Decision Task

Concerning accuracy, omissions appear to be null in the present experiment, potentially because the maximum cut-off response time was rather long (i.e, fixed to 5 s). In the visual modality, no significant effect was observed, i.e, the number of errors was equal between groups, *F*(2, 50) = 0.78, *p* = 0.46, and η^2^*_p_* = 0.03, and lexicality status, *F*(1, 50) = 1.18, *p* = 0.28, and η^2^*_p_* = 0.02. In the auditory modality, a lonely main effect of lexicality, *F*(1, 50) = 4.88, *p* = 0.03, and η^2^*_p_* = 0.09, i.e, more errors for pseudo-words than for words is observed, but Tukey HSD *post-hoc* testing failed to show any significant effect within groups.

#### Patients *off* Medication vs. Controls

In the visual modality, we observed a main effect of lexicality, with longer RTs for pseudowords (mean RT = 791 ± 149 ms) than for real words (mean RT = 688 ± 108 ms), *F*_1_(1, 33) = 40.6, *p* < 0.001, and η^2^*_p_* = 0.552 and *F*_2_(1, 58) = 188.4, *p* < 0.001, and η^2^*_p_* = 0.765. This main effect was statistically significant in both groups. The ANOVA also revealed a main effect of *g*roup, *F*_1_(1, 33) = 5.2, *p* = 0.02, and η^2^*_p_* = 0.152 and *F*_2_(1, 58) = 101.0, *p* < 0.001, and η^2^*_p_* = 0.635, and a lexicality ^∗^ group interaction, *F*_1_(1, 33) = 5.6, *p* = 0.03, and η^2^*_p_* = 0.135 and *F*_2_(1, 58) = 19.8, *p* < 0.001, and η^2^*_p_* = 0.247. The Tukey HSD *post hoc* test revealed a significant difference between groups for pseudowords (*p*_B__onferroni_ = 0.018), but not for words (*p*_B__onferroni_ = 1.): the mean value of the lexicality effect was greater in the PD group (146 ± 126 ms) than in the control group (68 ± 64 ms).

In the auditory modality, we also observed a significant main effect of lexicality in both groups, with longer RTs for pseudowords (mean RT = 1031 ± 143 ms) than for real words (mean RT = 909 ± 112 ms), *F*_1_(1, 33) = 34.4, *p* < 0.001, and η^2^*_p_* = 0.503 and *F*_2_(1, 58) = 59.6, *p* < 0.001, and η^2^*_p_* = 0.439. There was no main effect of *g*roup, *F*_1_(1, 33) = 0.0, *p* = 0.98, and η^2^*_p_* = 0.000 and *F*_2_(1, 58) = 0.0, *p* = 0.96, and η^2^*_p_* = 0.000, and no lexicality ^∗^ group interaction, *F*_1_(1, 33) = 1.7, *p* = 0.20, and η^2^*_p_* = 0.047 and *F*_2_(1, 58) = 10.2, *p* = 0.002, and η^2^*_p_* = 0.020 (Table 2).

**TABLE 2:** Mean reaction times (±standard deviation) in ms for word and pseudoword responses, for age-matched controls (CO) and patients with PD *on* or *off* medication, in the lexical decision task (LDT) and simple reaction time task (SRTT) in the visual and auditory modalities.

		VISUAL		AUDITORY	
		Words	Pseudowords	Words	Pseudowords
LDT	CO	663 (± 116)	731 (± 103)	890 (± 135)	1031 (± 165)
	PD *off*	718 (± 92)	864 (± 167)	926 (± 77)	1016 (± 104)
	PD *on*	685 (± 122)	783 (± 155)	872 (± 89)	988 (± 116)
SRTT	CO	272 (± 65)	270 (± 65)	385 (± 145)	402 (± 144)
	PD *off*	313 (± 67)	340 (± 97)	506 (± 133)	520 (± 150)
	PD *on*	300 (± 72)	304 (± 70)	424 (± 149)	450 (± 159)

#### Patients *on* Medication vs. Controls

We observed a main effect of lexicality in the visual modality, with longer RTs for pseudowords (mean RT = 756 ± 132 ms) than for real words (mean RT = 674 ± 118 ms), *F*_1_(1, 35) = 53.85, *p* < 0.001, and η^2^*_p_* = 0.606 and *F*_2_(1, 58) = 93.6, *p* < 0.001, and η^2^*_p_* = 0.617. This main effect was statistically significant in both groups. The ANOVA did not reveal a main effect of group, *F*_1_(1, 35) = 0.362, *p* = 0.362, and η^2^*_p_* = 0.024 and *F*_2_(1, 58) = 44.2, *p* < 0.001, and η^2^*_p_* = 0.432, or lexicality ^∗^ group interaction, *F*_1_(1, 35) = 1.83, *p* = 0.185, and η^2^*_p_* = 0.050 and *F*_2_(1, 58) = 8.40, *p* = 0.005, and η^2^*_p_* = 0.126.

We observed a significant main effect of lexicality in the auditory modality for both groups, with longer RTs for pseudowords (mean RT = 1017 ± 147 ms) than for words (mean RT = 886 ± 116 ms), *F*_1_(1, 35) = 47.48, *p* < 0.001, and η^2^*_p_* = 0.576 and *F*_2_(1, 58) = 71.1, *p* < 0.001, and η^2^*_p_* = 0.551. There was no main effect of group, *F*_1_(1, 35) = 0.624, *p* = 0.435, and η^2^*_p_* = 0.018 and *F*_2_(1, 58) = 30.0, *p* < 0.001, and η^2^*_p_* = 0.341, or lexicality ^∗^ group interaction, *F*_1_(1, 35) = 0.459, *p* = 0.502, and η^2^*_p_* = 0.013 and *F*_2_(1, 58) = 1.89, *p* = 0.174, and η^2^*_p_* = 0.032 (Table 2).

### Simple Reaction Time Task

#### Patients *off* Medication vs. Controls

In the visual modality, we observed main effects of lexicality, *F*_1_(1, 33) = 6.14, *p* = 0.018, and η^2^*_p_* = 0.153 and *F*_2_(1, 58) = 5.06, *p* = 0.028, and η^2^*_p_* = 0.031, and group, *F*_1_(1, 33) = 4.80, *p* = 0.035, and η^2^*_p_* = 0.124 and *F*_2_(1, 58) = 51.09, *p* < 0.001, and η^2^*_p_* = 0.278. There was also a lexicality ^∗^ group interaction, *F*_1_(1, 33) = 7.93, *p* = 0.008, and η^2^*_p_* = 0.189 and *F*_2_(1, 58) = 4.55, *p* = 0.037, and η^2^*_p_* = 0.025). The Tukey HSD *post hoc* test showed a significant difference between groups for pseudowords (*p*_B__onferroni_ = 0.005), but not for words (*p*_B__onferroni_ = 0.786). There was a lexicality effect of 27 ms in the PD group, but not in the control group (Table 2).

In the auditory modality, there was no main effect of lexicality, *F*_1_(1, 33) = 3.73, *p* = 0.07, and η^2^*_p_* = 0.099 and *F*_2_(1, 58) = 1.23, *p* = 0.273, and η^2^*_p_* = 0.021, and the lexicality ^∗^ group interaction was not significant, *F*_1_(1, 33) = 0.0, *p* = 0.094, and η^2^*_p_* = 0.000 and *F*_2_(1, 58) = 0.00, *p* = 0.969, and η^2^*_p_* = 0.000. We did, however, observe a main effect of group, *F*_1_(1, 33) = 5.82, *p* = 0.021, and η^2^*_p_* = 0.146 and *F*_2_(1, 58) = 57.04, *p* < 0.001, and η^2^*_p_* = 0.496, as patients were slower (513 ± 140 ms) than controls (394 ± 143 ms).

#### Patients *on* Medication vs. Controls

In the visual modality, there was no main effect of either lexicality, *F*_1_(1, 35) = 0.023, *p* = 0.881, and η^2^*_p_* = 0.001 and *F*_2_(1, 58) = 0.042.94, *p* = 0.837, and η^2^*_p_* = 0.001, or group, *F*_1_(1, 35) = 2.26, *p* = 0.141, and η^2^*_p_* = 0.061 and *F*_2_(1, 58) = 41.94, *p* < 0.001, and η^2^*_p_* = 0.420. There was no lexicality ^∗^ group interaction, *F*_1_(1, 35) = 0.148, *p* = 0.703, and η^2^*_p_* = 0.004 and *F*_2_(1, 58) = 0.019, *p* = 0.891, and η^2^*_p_* = 0.000.

In the auditory modality, we found no effect of lexicality, *F*_1_(1, 35) = 8.35, *p* = 0.007, and η^2^*_p_* = 0.193 and *F*_2_(1, 58) = 1.35, *p* = 0.250, and η^2^*_p_* = 0.023. The lexicality ^∗^ group interaction was not significant, *F*_1_(1, 35) = 0.061, *p* = 0.080, and η^2^*_p_* = 0.002 and *F*_2_(1, 58) = 0.89, *p* = 0.766, and η^2^*_p_* = 0.002. There was no main effect of group, *F*_1_(1, 35) = 0.0434, *p* = 0.514, and η^2^*_p_* = 0.012 and *F*_2_(1, 58) = 7.53, *p* = 0.008, and η^2^*_p_* = 0.115 (Table 2).

## Discussion

The goal of the present study was to investigate motor execution and lexical access in patients with PD, *on* or *off* medication, and age-matched controls. Besides well documented motor symptoms, recent studies have highlighted cognitive impairments in patients with PD. However, a possible impairment of linguistic processes (e.g., access to mental lexicon) has seldom been investigated in PD. To determine which processes (perceptual, motor, or linguistic) might be affected in patients when it comes to lexical access, we used two tasks that differed on the cognitive/linguistic processes they elicit: an SRTT in which participants simply had to respond as quickly as possible when the stimulus appeared, whatever its lexical status (word or pseudoword), and an LDT, where they had to decide whether the stimulus was a real word or a pseudoword. The SRTT gives an estimate of the temporal costs of perceptual and motor processes, independently of any linguistic features. In the LDT, additional temporal costs are generated by the lexical processing of the stimuli. Within this general word recognition framework, we administered the tasks in either a visual or an auditory modality, to determine whether none, one or both types of perceptual input are modulated in PD.

After discussing the results of the preliminary experiment conducted among young participants to validate our methodological choices (e.g., tasks, stimuli), we discuss the comparison between patients *off* medication and controls, starting with the most peripheral (i.e., motor and sensory) aspects, then the cognitive-linguistic ones. We then compare patients *on* medication and controls. We end by identifying several limitations of this study.

### Experimental Validation in Young Participants

Before the main experiment conducted among patients with PD and age-matched controls, we ran a validation experiment in which we tested the stimuli we had created among young adults, who are usually recruited as participants in studies such as ours. A total of 40 participants therefore underwent both tasks (LDT and SRT) in the same order as the older participants, and with the same visual and auditory stimuli.

In the SRTT, as expected, the young participants responded just as quickly for words as they did for pseudowords: no lexicality effect was observed. Auditory stimuli gave rise to slightly longer RTs (+47 ms) than visual stimuli did, probably because the onset of visual stimuli was instantaneous, whereas more time was needed to detect the onset of the auditory stimuli.

In the LDT, RTs were longer than they were for the SRTT, the additional duration (480 ms) corresponding to the time needed for lexical access and decision making. Once again, RTs were longer for the auditory modality (836 ms) than for the visual one (609 ms), as the word or pseudoword had to be listened to until the offset (mean duration: approx. 500 ms) before a lexical decision could be made. A lexicality effect was expected and observed in the LDT. This effect was of equal duration in both modalities (about 70 ms), as the decision-making process was the same. Taken together, these results in young participants validated the methodology we used in our experiment, in terms of both stimulus construction and protocol design.

### Motor Deficits in Patients With PD?

The SRTT is a relevant means of estimating possible motor deficits (akinesia and bradykinesia) in patients with PD, as it requires very few cognitive resources. In the visual modality, all the patients responded to real words as quickly as controls. These fast responses suggest that their performance was not hindered by bradykinesia. Interestingly, this may seem to run counter to descriptions in the literature (Gauntlett-Gilbert and Brown, 1998; Favre et al., 2013), as increased RTs attributed to akinesia have often been reported in patients with PD (Evarts et al., 1981). However, this effect has not been systematically observed, and probably depends on several parameters, in particular, patients’ age and age at onset of the disease (Reid et al., 1989; Fimm et al., 1994), and the presence/absence of bradyphrenia (Mayeux et al., 1987). Patients’ slowdown is also related to deficits in attentional processes (Goodrich et al., 1989). The results of the present study confirm that motor execution *per se* is not systematically slowed in patients with PD either *off* or *on* medication, especially not in the kinds of task we used here.

### Hearing Deficits in Patients With PD *off* Medication?

In the auditory modality, the SRTT revealed longer RTs in patients with PD *off* medication, compared with control participants. Since this was not the case in the visual modality, in which patients responded as quickly as controls, this slowness responding to auditory stimuli suggests that patients have hearing loss, compared with age-matched controls. Specific hearing loss has recently been recognized as an additional nonmotor feature in patients with PD (Vitale et al., 2012), even in *de novo* patients (Pisani et al., 2015). From a pathophysiological point of view, the natural aging process, combined with the intrinsic neurodegenerative changes in PD, could interfere with cochlear transduction mechanisms, contributing to presbycusis (Vitale et al., 2012). However, we did not specifically measure participants’ hearing acuity, and further research is required to elucidate the involvement of an auditory perceptual deficit in PD in higher-order language processes.

### Inhibition Deficits in Patients With PD *off* Medication?

Patient groups both *off* and *on* medication had significantly lower MDRS scores than controls, as previously observed (Schmidt et al., 1994; McDermott et al., 2018). When we set an MDRS cut-off score of <140/144 for PD with mild cognitive impairment, in line with Matteau et al. (2012), a total of 58% of patients *on* medication and 81% of patients *off* medication fell within this category. This confirmed that the MDRS is a sensitive instrument for evaluating the general decrease in cognitive functioning in PD (Kulisevsky and Pagonabarraga, 2009), but lacks sufficient specificity to precisely estimate inhibitory ability. Inhibition deficits have already been reported in patients with PD (Gauggel et al., 2004; Favre et al., 2013) as part of a more global executive dysfunction (for a review, see Dirnberger and Jahanshahi, 2013).

In our study, results on both SRTT and LDT pointed to inhibition deficits in patients *off* medication. In the SRTT in the visual modality, RTs for pseudowords, but not real words, were significantly longer in the PD *off* medication group than in the control group, inducing an unexpected but significant lexicality effect. This effect could be interpreted as reflecting patients’ difficulty inhibiting irrelevant processing. An alternative interpretation is that patients had difficulty switching from the LDT to the SRTT, and therefore incorrectly applied the strategy used for the first task to the second task. This is a plausible interpretation, as patients with PD have been shown to have difficulty switching from one task to another (Witt et al., 2006; Cameron et al., 2010). Nevertheless, it can be ruled out in the present case, for if patients had applied the same strategy in the SRTT as they had done in the LDT, their RTs would have been much longer. As it was, their RTs (∼300 ms) were fully compatible with those expected in an SRTT and comparable to those of controls for real words. We therefore think that the problem came from elsewhere and was specific to pseudowords.

The visual presentation of a word is known to automatically trigger access to the mental lexicon (McClelland and Rumelhart, 1981). Event-related potential studies have shown that this process can take place very rapidly after the presentation of the visual stimulus (∼100 ms), and the detection of word/pseudoword differences occurs just 160 ms after stimulus onset (e.g., Hauk et al., 2006). Some cognitive resources are allocated to this automatic processing, and when the task requires the inhibition or deactivation of this processing, additional resources are required. It is therefore likely that patients *off* medication struggled to inhibit the reading of the items in the SRTT. This slowdown is reminiscent of the classic Stroop effect (Stroop, 1935), in which irrelevant information interferes with the performance of a cognitive task. Similar interpretations have previously been proposed (Taylor et al., 1986; Gotham et al., 1988), whereby patients with PD have difficulty ignoring irrelevant information or inhibiting its processing (Hietanen and Teräväinen, 1988; Henik et al., 1993).

For the SRTT in the auditory modality, patients did not exhibit a lexicality effect, but their RTs (around 500 ms) suggest that they made their manual responses before the end of the auditory stimulus, when they did not yet know what the latter was. They presumably accessed the mental lexicon too late for it to slow down their response. This may explain why no lexicality effect was observed in the auditory modality, contrary to the visual modality in which lexical access was very fast because the word was instantaneously displayed.

For the LDT in the visual modality, patients *off* medication had slower RTs than control participants for pseudowords, but not for real words. Patients therefore exhibited a greater lexicality effect than controls, whereas Marí-Beffa et al. (2005) reported similar lexicality effects in both groups. As mentioned earlier, pseudowords have not always been treated as stimuli of interest in lexical decision studies. Rather, they have often been regarded as mere fillers, and the linguistic processes subtending their processing have rarely been modelled. According to the conventional dual-route model of reading (Morton and Patterson, 1980; Coltheart et al., 2001), the identification of the lexical status of the stimuli depends on the activation of the direct (lexical) pathway for words, which is faster than the indirect (sublexical) pathway used for the recognition of pseudowords, which requires grapheme-phoneme conversion. This implies the lateral inhibition of competitors (McClelland and Rumelhart, 1981). The more similar the words in the lexicon, the greater the competition between them and the slower the response. We can guess that, owing to the close orthographic proximity of the two kinds of stimuli in this study, pseudowords also activated similar neighboring words. In patients, this activation turned into overactivation because of the deficit/dysfunction of the process needed to inhibit competitive words, and therefore slowed down the responses of patients more than controls. Focusing on the idea of competition and lateral inhibition between ambiguous words (Watters and Patel, 2002), Gurd and Oliveira (1996) showed that patients with PD have difficulty choosing an appropriate word from a list of semantically competitive words.

This hypothesis also fits with the computational modelling of lexical decision, which tries to determine how participants respond negatively when the stimulus is not a real word (Dufau et al., 2012). The leaky competing accumulator model of the LDT, derived from the multiple read-out model (Grainger and Jacobs, 1996), represents an alternative way of understanding lexical decision mechanisms (Usher and McClelland, 2001; Dufau et al., 2012). In this model, a *no* response is generated if insufficient evidence for a *yes* response has been accumulated before the deadline is reached. It is composed of yes and no nodes, both activated constantly and equally before the trial. In the absence of any evidence for a nonword, the no response node is equal to the constant total input value minus the evidence for a real word extracted from the stimulus. This model also features mutually inhibitory connections between the two response nodes, such that a rise in activity in one automatically causes a reduction in activity in the other, and vice versa. From this point of view, and as mentioned above, the patients in our experiment may have had difficulty correctly inhibiting the *yes* response when a *no* response was needed.

Concerning the LDT in the auditory modality, the participants’ RTs were much longer than they were in the visual modality, owing to the need to hear enough auditory information to perform the task. The expected lexicality effect was observed whatever the group. Patients *off* medication did not exhibit any difficulty with phonological processing, and performed similarly to controls. We can conclude that, owing to the slow processing of the auditory signal, this experimental condition is not ideal for revealing difficulty with the cognitive-linguistic processes involved in lexical access.

### Comparison Between Patients *on* Medication and Age-Matched Controls

Overall, in both tasks and both sensory modalities, the performances of patients *on* medication were no different from those of age-matched controls. As was already the case in patients *off* medication, no bradykinesia was noted in their responses to the SRTT. In addition, and contrary to the patients *off* medication, no unexpected lexicality effect was exhibited in the visual modality, and no slowness in the auditory modality, compared with controls. Therefore, patients *on* medication and controls did not differ on the motor and auditory processes elicited by the SRTT. Finally, in the LDT, the magnitude of the lexicality effect was no different from that of controls. We may thus conclude that dopaminergic medication was able to restore motor, perceptual and cognitive functioning close to normal in the patients with PD in the present study. However, to confirm this medication effect and draw a more robust conclusion, a further study involving a single set of patients tested both *off* and *on* medication is required, as confounding factors (education, sex, and verbal IQ, etc.) may have influenced the results in the present between-participants experimental design.

## Limitations of the Study

Several limitations have already been mentioned in specific parts of the Discussion. An additional one is the small sample size, as this reduced the power and generalizability of the results. As already mentioned, the same participants with PD should have been tested both *on* and *off* medication, to precisely evaluate its effect. Finally, patients’ hearing should probably be performed systematically before any study featuring sound stimuli.

## Conclusion

We found that motor execution *per se* was not slowed in patients with PD either *on* or *off* medication, as they were just as fast as controls in the visual modality when the stimuli were real words. At the sensory level, however, the hearing acuity of patients *off* medication seemed to be deficient, compared with that of the age-matched controls and patients *on* medication. In addition, the unmedicated patients were slower than controls when the stimuli were pseudowords, even when the task (SRTT) did not require them to differentiate between the stimuli. Finally, the classic lexicality effect was of the same magnitude in patients *on* medication and controls, but amplified in PD patients *off* medication. We conclude that patients with PD have difficulty inhibiting a cognitive-linguistic process (i.e., reading) when not necessary (SRTT) and exhibit a particular deficit in pseudoword processing, which may be related to impaired lateral word inhibition within the mental lexicon. This raises the question of whether this lack of inhibition is specific to lexical processing, or whether it reflects a more general deficit that affects other types of linguistic features. The basal ganglia are acknowledged to be key substrates of high-level cognitive domains. This role reflects their complex organization and multiple circuitries, including pathways through cortico-subcortical loops (Leh et al., 2007; Haber and Calzavara, 2009). More specifically, a network involving the basal ganglia, thalamus, and Broca’s area is involved in language processing (Ford et al., 2013; see also Moro et al., 2001; Crosson et al., 2003). However, it has yet to be ascertained whether language impairments following basal ganglia damage are primary or epiphenomenal to other cognitive dysfunctions, and further dedicated studies are therefore needed in this field.

## Data Availability Statement

The raw data supporting the conclusions of this article will be made available by the authors, without undue reservation.

## Ethics Statement

The studies involving human participants were reviewed and approved by Ethical Research Committee Sud Méditerranée 1, France. The patients/participants provided their written informed consent to participate in this study.

## Author Contributions

AL, J-LV, FV, and SP designed the study. AL performed the data acquisition. FV was in charge of patient recruitment and performed all the clinical assessments. AL performed statistical analyses of the data. AL, J-LV, and SP analyzed, interpreted, and drew conclusions from the results. AL wrote the first draft of the manuscript. J-LV, FV, and SP revised and participated in the writing of the article. All the authors read and approved the final draft.

## Conflict of Interest

The authors declare that the research was conducted in the absence of any commercial or financial relationships that could be construed as a potential conflict of interest.

## Footnotes

1 From a psycholinguistics point of view, the mental lexicon is a set of representations of words known by an individual. It is a highly organized system, where units are linked together according to their phonological, orthographic and semantic similarities. *Lexical access* describes the cognitive operations that take place when a stimulus activates the lexical representation of a word, allowing it to be recognized (Spinelli and Ferrand, 2005).

2 Coltheart et al. (2001) later proposed an alternative version of this model, in which the system involves cascading rather than binary (*all or nothing*) processing: the dual-route cascaded (DRC) model (Coltheart et al., 2001). This model has three main stages: (1) word activation is gradual and not all or nothing as it was in the earlier version (Coltheart, 1978); (2) the processing takes place in cascade, where word reading involves the parallel activation of both the direct and indirect routes; and (3) processing is interactive, at least that of the direct channel, meaning that each module is bidirectionally connected with its adjacent modules. According to the DRC model, the direct route is composed of a visual recognition system and a phonological one.
