# Supplementary material for: Altered Inhibitory Mechanisms in Parkinson’s Disease: Evidence From Lexical Decision and Simple Reaction Time Tasks
Source: Front Hum Neurosci. 2021 Apr 26;15:624026. doi: 10.3389/fnhum.2021.624026 (PMC8107209; doi:10.3389/fnhum.2021.624026)
Supplement: Supplementary file 1 [file Table_1.DOCX]

| **Words**  [English traduction] | **Number of phonemes** | | **Number of graphemes** | **﻿Number of phonological AND orthographic neighbors** | **Frequency** | **First syllable frequency** | **Second syllable frequency** | **Pseudo-words** |  |
| --- | --- | --- | --- | --- | --- | --- | --- | --- | --- |
| baron [baron] | 4 | 4 | | 1 | 7.58 | 1257.37 | 365.51 | batin |  |
| bilan [appraisal] | 4 | 4 | | 0 | 12.95 | 348.24 | 295.30 | dipin |  |
| bocal [jar] | 5 | 5 | | 2 | 28.34 | 259.09 | 142.01 | bolan |  |
| butin [plunder] | 4 | 4 | | 7 | 2.66 | 231.61 | 956.12 | bulon |  |
| canal [channel] | 5 | 5 | | 3 | 14.83 | 2800.19 | 379.98 | cavan |  |
| colis [package] | 4 | 5 | | 23 | 15.94 | 3454.17 | 706.08 | colut |  |
| coton [cotton] | 4 | 4 | | 0 | 27.33 | 3454.17 | 577.10 | cotin |  |
| culot [nerve] | 4 | 5 | | 5 | 0.76 | 163.44 | 385.07 | cupos |  |
| divan [sofa] | 4 | 4 | | 0 | 2.04 | 4641.69 | 2146.56 | bilet |  |
| filet [net] | 4 | 5 | | 23 | 46.53 | 1157.68 | 1131.44 | fibot |  |
| gilet [vest] | 4 | 5 | | 47 | 14.37 | 169.66 | 1131.44 | gipas |  |
| jeton [token] | 4 | 4 | | 0 | 0.96 | 6848.82 | 577.10 | jelon |  |
| lapin [rabbit] | 4 | 4 | | 83 | 177.31 | 28350.08 | 433.73 | lalot |  |
| maman [mom] | 4 | 4 | | 0 | 774.92 | 4637.92 | 6917.15 | mafus |  |
| matin [morning] | 4 | 4 | | 79 | 474.82 | 4637.92 | 956.12 | mapin |  |
| melon [melon] | 4 | 4 | | 135 | 12.15 | 2299.74 | 828.03 | meban |  |
| mulet [mule] | 4 | 5 | | 5 | 1.83 | 424.83 | 1131.44 | muton |  |
| ravin [ravine] | 4 | 4 | | 6 | 3.48 | 1665.20 | 129.74 | racal |  |
| refus [refusal] | 4 | 5 | | 0 | 2.99 | 6103.79 | 28.50 | relon |  |
| repas [meal] | 4 | 5 | | 33 | 178.50 | 6103.79 | 187.00 | reman |  |
| repos [rest] | 4 | 5 | | 179 | 33.11 | 6103.79 | 447.64 | reron |  |
| robot [robot] | 4 | 5 | | 2 | 77.45 | 360.19 | 265.48 | rolet |  |
| ruban [tape] | 4 | 4 | | 0 | 26.80 | 499.27 | 52.18 | rulis |  |
| salon [living room] | 4 | 4 | | 180 | 53.47 | 5160.45 | 828.03 | sanal |  |
| salut [greeting] | 4 | 5 | | 17 | 20.07 | 5160.45 | 249.58 | sanin |  |
| sapin [fir tree] | 4 | 4 | | 184 | 77.63 | 5160.45 | 433.73 | saton |  |
| savon [soap] | 4 | 4 | | 53 | 37.34 | 5160.45 | 387.16 | savin |  |
| talon [heel] | 4 | 4 | | 53 | 7.80 | 1223.68 | 828.03 | talet |  |
| valet [servant] | 4 | 5 | | 11 | 5.28 | 1744.75 | 1131.44 | vavon |  |
| venin [venom] | 4 | 4 | | 0 | 3.90 | 697.73 | 111.43 | velet |  |
|  |  |  | |  |  |  |  |  |  |

Table : Linguistic characteristics of the items issues from Manulex (Lété, Sprenger-Charilles, Colé, 2004)

# References

Lété, B., Sprenger-Charolles, L., & Colé, P. (2004). MANULEX: A grade-level lexical database from French elementary school readers. *Behavior Research Methods, Instruments, & Computers*, *36*(1), 156-166.
