# Supplementary material for: Altered Inhibitory Mechanisms in Parkinson’s Disease: Evidence From Lexical Decision and Simple Reaction Time Tasks
Source: Front Hum Neurosci. 2021 Apr 26;15:624026. doi: 10.3389/fnhum.2021.624026 (PMC8107209; doi:10.3389/fnhum.2021.624026)
Supplement: Supplementary file 2 [file Table_2.DOCX]

Table : Medication characteristics and Levodopa equivalence daily doses (LEDD, Tomlinson et al., 2010; Shade, Mollenhauer & Trenkwalder, 2020) of ON and OFF participants.

|  | **PD off medication** | |  | **PD on medication** | |
| --- | --- | --- | --- | --- | --- |
|  | **Daily medication** | **LEDD (mg)** |  | **Daily medication** | **LEDD (mg)** |
| 1 | Levodopa (200mg)*3 + Pramipexole (1.05mg)*1 + Rasagiline (1mg)*1 | 805 | 1 | Levodopa (100mg)*3 | 300 |
| 2 | Levodopa (250mg)*3 + Pramipexole ER (0.56mg) | 806 | 2 | Levodopa (200mg)*3 + Entacapone (200mg)*3 | 798 |
| 3 | Levodopa (250mg)*3 + Rasagiline (1mg)*1 | 850 | 3 | Levodopa (250mg)*3 + Piribedil ER (50mg)*3 | 900 |
| 4 | Levodopa (100mg)*4.5 | 450 | 4 | Levodopa (150mg)*2 + Levodopa (200mg)*3 + Entacapone (200mg)*5 + Pramipexole (2.1mg)*1 + Pramipexole (1.05mg)*2 + Rasagiline (1mg)*1 | 1750 |
| 5 | Levodopa (100mg)*3 + Levodopa (75mg)*4 + Entacapone (200mg)*4 + Piribedil ER (50mg)*3 | 1014 | 5 | Levodopa (250mg)*2 | 500 |
| 6 | Levodopa (250mg)*6 | 1500 | 6 | Levodopa (150mg)*2 + Levodopa (200mg)*1 + Levodopa (150mg)*1 + Entacapone (200*2*0,33) + Amantadine (100mg)*2 | 982 |
| 7 | Rasagiline (1mg) | 100 | 7 | Levodopa (200mg)*1 + Entacapone (200mg)*1 + Pramipexole (2,1mg)*1 + Rasagiline (1mg)*1 + Amantadine (100mg)*1 | 676 |
| 8 | Levodopa (100mg)*6.5 | 650 | 8 | Levodopa (100mg)*2 + Pramipexole (2,1mg)*1 + Rasagiline (1mg)*1 | 510 |
| 9 | Levodopa (100mg)*2 + Ropinirole (8mg)*1 + Ropinirole (4 mg)*1 | 440 | 9 | Levodopa (50mg)*5 + Levodopa (200mg)*5 + Entacapone (200mg)*5 + Rasagiline (1mg) | 1680 |
| 10 | Levodopa (150mg)*3 + Entacapone (200mg)*3 + Rasagiline (100mg)*1 | 748 | 10 | Rotigotine (8mg)*1 | 240 |
| 11 | Levodopa (250mg)*3 + Piribedil ER (50mg)*3 | 900 | 11 | Pramipexole ER (2,1mg)*1 + Rasagiline (1mg)*1 | 310 |
| 12 | Levodopa (100mg)*2 + Levodopa (200mg)*4 + Entacapone (200mg)*4 + Ropinirole (8mg)*1 + Ropinirole (4mg)*1 | 1504 | 12 | Levodopa (100mg)*3 + Piribedil ER (50mg)*3 | 450 |
| 13 | Levodopa (75mg)*2 + Levodopa (50mg)*1 + Entacapone (200mg)*3 + Ropinirole (4mg)*1 + Ropinirole (2mg)*1 | 518 | 13 | Levodopa (100mg)*1 + Levodopa (100mg)*4 + Entacapone (200mg)*4 + Rasagiline (1mg)*1 | 864 |
| 14 | Levodopa (250mg)*5 + Rasagiline (1mg)*1 | 1350 | 14 | Levodopa (250mg)*3 + Pramipexole (0,7mg)*3 + Amantadine (100mg)*3 | 1260 |
| 15 | Levodopa (200mg)*3 | 600 | 15 | Piribedil ER (50mg)*1 + Rasagiline (1mg)*1 + Rotigotine (16mg)*1 | 630 |
| 16 | Levodopa (150mg)*3 + Entacapone (200mg)*3 + Rasagiline (100mg)*1 | 748 | 16 | Levodopa (100mg)*6 + Rasagiline (1mg)*1 | 700 |
|  |  |  | 17 | Levodopa (100mg)*4 + Entacapone (200mg)*2 + Ropinirole ER (8mg)*2 + Ropinirole ER (4mg)*1 | 932 |
|  |  |  | 18 | Levodopa CR (200mg)*1 + Levodopa (100mg)*3 + Levodopa (50mg)*1 + Entacapone (200mg)*4 + Ropinirole ER (8mg)*1 + Rasagiline (1mg)*1 | 1024 |

# References

Tomlinson, C. L., Stowe, R., Patel, S., Rick, C., Gray, R., and Clarke, C. E. (2010). Systematic review of levodopa dose equivalency reporting in Parkinson’s disease. *Movement Disorders* 25, 2649–2653.

Schade, S., Mollenhauer, B., & Trenkwalder, C. (2020). Levodopa equivalent dose conversion factors: an updated proposal including opicapone and safinamide. *Movement disorders clinical practice*, *7*(3), 343-345.
